# Some Account of the Gums, Not Included among Those Called Fœtid

**Published:** 1809-11

**Authors:** 


### Some Account of the Gums, not included among those called Fœtid.

MOST of those concrete substances brought from abroad under the name of gums, contain a considerable quantity of resin in them. They are usually much encumbered with pieces of stalk, leaves, and often of common earth. To purify them from these, it is sometimes found sufficient to soften them by heat, and strain them; at other times, it is necessary to dissolve or rather diffuse them in a considerable quantity of water, and after straining them, again to inspissate them with heat. If the gum thus strained contains much resin, the quantity of water must be very great, and much pains must be taken, lest in cooling a separation should take place between the resin, which is more insoluble, and the gum. In all these operations there will be danger lest some of the volatile particles, which are often often the most efficient, should be dissipated. On this account those gums which are brought over in the original lachrymæ, or tears, are preferred.

Having in one of your former Numbers examined the fœtid gums, I shall now notice a few others which did not come under that description.

The Gum Arabic and Senega are perhaps more strictly gums than any other that pass under that name; that is, they are perfectly soluble in water, and indissoluble in spirit or oils, losing none of their properties by mixture in boiling water and subsequent evaporation, and burning to a black coal when submitted to fire, without melting or inflaming.

The properties of gum arabic are so generally known that we need make but few remarks upon it. It is admitted to be the produce of the Egyptian Acacia, Mimosa milotina of Linnæus. When gum arabic is dissolved in water, it will admit the addition of any acids, and even of saline mixtures; but if alkalies are added, a coagulation takes place, and the gum separates in clouds. The same happens if spirit is added. The mucilage of gum arabic is however extremely useful in forming a kind of emulsion with water and many substances which are not of themselves soluble in that fluid. Oils, resins, and balsams are readily broken and diffused by a proper mixture of gum, and even rendered palatable. But these emulsions are separated by those substances which coagulate the gummy solutions, particularly by the alkalies.

The gum Senega seems no way to differ from the gum arabic, excepting that its colour is more inclined to yellow, and it is oftener found with pieces of wood and other impurities. However, they are so much alike, that the clearest pieces of the Senega are often picked out and sold as the gum arabic.

Gum tragacanth partakes of most of the properties of the former gums, but can scarcely be called soluble in any menstruum. It thickens an almost incredible quantity of water, and if more is added, in order to render the mixture thin and transparent, the consequence is a separation of the particles; the gum falling in clouds to the bottom. It is the produce of the Astragalus tragacantha of Linnæus, and grows wild in warmer climates. Though it bears the cold of ours, it does not produce its gum, which is brought to us from Turkey in the form we see it.

Most, if not all the other gums, contain a considerable portion of resin, and some may be truly called resins.

The ammoniacum, is a gummy resinous juice brought from the East Indies, and from Egypt likewise. We are ignorant of the tree from which it is derived.

Rubbed carefully with water, it forms an emulsion, called in our dispensatories, Lac ammoniaci. On account of the gender of lac, we are too apt, from an indolent inattention, to call it lac ammoniacum, an error we should always avoid. These apparent trifles are so grating to classical ears, as often to lessen their opinion of those who use them.

Ammoniacum is a truly valuable remedy as a pectoral. It is not so much in use as it deserves, partly, on account of its disagreeable taste and smell. When given in pills, it is not found equally efficacious, probably, from its slow solution in the stomach, or from its passing off entire.

The gum Anime is reserved as a specimen, but its very contracted use renders it deserving of little notice. It is the produce of the Hymenæa courbaril of Linnæus, a native of the Brazils and New Spain. It is almost entirely soluble in spirit of wine, as is the gum Elemi, the produce of the tree called by Linnæus, the Anyris Elemifera, brought like the former from the Spanish West Indies.

This gum has a strong and not unpleasant smell; whether it has any medicinal virtues internally is not certainly known, but probably not. As an external application, it forms a considerable ingredient in the linimentum Arcæi, which has always been considered a good digestive ointment.

Gumboge is the produce of the Gambogia gutta of Linnæus; it is said to have derived its name from Cambodia, in the East Indies. Whether this is the case or not, it is certainly produced in other settlements of the East. It is much more an article in trade as a pigment than a medicine, affording a most beautiful yellow, which by an alkaline salt is changed into a florid blood red. As a medicine it is very powerful, but more uncertain in its operation than is convenient in an art abounding enough in uncertainties. It was once highly prized as an hydrogogue, but the violence of its operation, and its tendency to prove emetic, has very much lessened its use. With the addition of calomel, it is said to lose its emetic qualities; but when it passes the stomach, its cathartic powers are sufficient of themselves, without the addition of any other uncertain remedy

The Guaiacum is the resinous gum of a tree called by Linnæus Guaiacum officinales. It grows principally in

Hispaniola,

Hispaniola, but is found also in Jamaica and some other West India islands. This concrete resinous substance is the inspisated juice exuding from incisions made in the trunk. It is brought over in irregular masses of a dusky green, and sometimes reddish. There is a mixture of gummy matter, which seems to contain none of the properties of the resin, which last is easily separated by spirit of wine. The virtues of gum guaiacum are much greater than they are usually estimated. Our excessive fondness for novelty has made us overlook many well-known remedies, and none more than guaiacum. In chronic rheumatism, more particularly, those troublesome local affections, known by the names of sciatica and lumbago, the guaiacum is a truly valuable remedy. It should always be given in doses sufficient to purge, and even freely where the constitution will bear it, which is generally the case. I have seen delicate subjects bear a very violent operation from this remedy, and feel considerable relief. It is neither my wish, nor would it be proper on this occasion to enter into any inquiry into the modus operandi; but I could almost say, that gum guaicum proves at once purgative, cordial, and strengthening, relieving pains in those deep seated parts which are hardly affected but by this and similar remedies. If it has fallen into disgrace, it has been by those who have employed it in acute or highly inflammatory cases of rheumatism. In these, the only relief we can expect from it is its purgative quality, which is not always induced, and where it is, other purgatives are preferable, in addition to every other antiphlogistic remedy. This error in applying the same remedy to the acute aud chronic state of a disease is not confined to guaiacum or rheumatism. It is much too common in a variety of other diseases; but in all, the practice marks the lowest degree of empiricism. The acute and chronic gout require treatments as different as the plethora of the juvenile Dives, and the emaciation of Lazarus.

The Kino is a gum but lately introduced into medicine. For many years it was, in familiar language, called Dr. Fothergill's gum, on account of a paper of that candid and industrious physician in the London Med. Obs. and Enquiries. It is there called Gummi rubrum astringens gambiense. Kino, in the Edinburgh Pharmacopœia. In the New London Pharmacopœia it is called Arboris nondrum descriptæ Afrianæ gummi-resina. This gum, as far as its chemical properties may be depended on, contains besides the two principles of mucilage and resin, a peculiarly astringent gent property, by which its solution, whether in water or spirit, may be entirely precipitated, so as to leave the liquor limpid, and almost pure. Acids of any kind render the solution turbid, and produce a precipitate. However, by further experiments it is proved, that the astringent or colouring matter is more completely taken up by spirit than by water.

The virtues of kino are not yet sufficiently ascertained, to determine the rank it should hold among the astringents. As far as we can learn by its sensible properties, it must be considered worthy of attention. In common practice, we know but little of dysentery, the dreadful disease of camps, and among the unsheltered poor of less fortunate countries. Whether it has any powers beyond the Catechu or Terra japonica, as it is usually called, I pretend not to determine.

